# Imaging Modalities in Craniosynostosis: A Systematic Review and Proposal of the ARCANA Protocol for Multimodal Radiation-Free Assessment

**DOI:** 10.3390/diagnostics15202632

**Published:** 2025-10-18

**Authors:** Mirko Micovic, Bojana Zivkovic, Ivan Vukasinovic, Drago Jelovac, Milan Stojicic, Vladimir Bascarevic

**Affiliations:** 1 Clinic of Neurosurgery, University Clinical Center of Serbia, Dr Koste Todorovica Street 4, 11000 Belgrade, Serbia; mvmicovic@gmail.com (M.M.); zivkovicbojanamd@gmail.com (B.Z.); 2 Faculty of Medicine, University of Belgrade, Dr Subbotica Street 8, 11000 Belgrade, Serbia; milan.stojicic@gmail.com; 3 Center for Radiology, University Clinical Center of Serbia, Pasterova 2, 11000 Belgrade, Serbia; vukasinovic_i@yahoo.co.uk; 4 Clinic for Maxillofacial Surgery, University Clinical Center of Serbia, Pasterova 2, 11000 Belgrade, Serbia; jbdrago@gmail.com; 5 Faculty of Dental Medicine, University of Belgrade, 11000 Belgrade, Serbia; 6 Clinic for Burns Plastic and Reconstructive Surgery, University Clinical Center of Serbia, Pasterova 2, 11000 Belgrade, Serbia

**Keywords:** computed tomography, stereophotogrammetry, ultrasound, magnetic resonance imaging

## Abstract

**Background/Objective:** Craniosynostosis, the premature fusion of one or more cranial sutures, is the second most common craniofacial defect and poses significant diagnostic and therapeutic challenges. Our objective was to systematically evaluate current diagnostic imaging modalities for craniosynostosis and to propose a novel radiation-free ARCANA Protocol as an alternative to conventional screening. **Methods:** Following PRISMA guidelines, we conducted a systematic review of the literature using PubMed and Cochrane databases from 2015 onwards, restricted to English-language and full-text articles. Inclusion criteria encompassed studies evaluating diagnostic accuracy, radiation exposure, and neurocranial outcomes associated with imaging modalities in craniosynostosis. Quality assessment was performed using QUADAS-2. To evaluate the certainty of evidence supporting each imaging modality, we applied the GRADE framework. Given the extensive number of included studies (*n* = 70), findings were categorized by diagnostic modality rather than individual studies. **Results:** Analysis of 70 selected studies demonstrated a continued reliance on 3D computed tomography (3DCT) as the diagnostic gold standard, despite recognized risks of cumulative radiation exposure in pediatric populations. Alternative radiation-free imaging techniques including high-resolution ultrasonography (US), three-dimensional stereophotogrammetry (3DSPG), and advanced magnetic resonance imaging (MRI) have emerged, offering substantial benefits such as eliminating ionizing radiation and providing comprehensive neurocranial assessments. 3DCT demonstrates approximately 90% sensitivity and 90–100% specificity for detecting suture closure; ultrasound achieves 71–100% sensitivity and 86–100% specificity, while advanced MRI techniques such as GA-VIBE report up to 97% sensitivity and 96% specificity. **Conclusions:** The proposed ARCANA Protocol integrates clinical assessment, 3DSPG, US, and advanced MRI sequences into a unified multimodal framework that eliminates radiation exposure while ensuring comprehensive evaluation of cranial and intracranial anatomy. The protocol emphasizes patient safety and diagnostic accuracy. The main limitations of this study are the heterogeneity of the included studies and the lack of prospective validation, which is essential to confirm diagnostic and clinical effectiveness and to support a potential paradigm shift toward radiation-free assessment of craniosynostosis.

## 1. Introduction

Craniosynostosis (CS) is defined as the premature fusion of one or more major cranial sutures, affecting approximately one in 2500 live births and standing as the second most prevalent craniofacial defect [1,2]. Beyond its morphological manifestations, CS is increasingly recognized as a neurodevelopmental condition wherein early sutural fusion restricts perpendicular skull growth, potentially leading to developmental delays and long-term neurocognitive implications [3,4]. Prompt and accurate diagnosis is therefore critical for timely surgical intervention.

Three-dimensional computed tomography (3DCT) is traditionally considered as the diagnostic gold standard for CS, providing rapid, high-resolution visualization of cranial sutures and osseous morphology. In modern setting, it is widely available and doesn’t require sedation. However, the continued reliance on ionizing radiation creates a well-recognized clinical dilemma in pediatric population, where children represent an exceptionally vulnerable group to radiation-induced effects [5,6,7,8]. Estimate dosage for pediatric head 3DCT is different for different age group, but considering that the most CS are diagnosed in early age (infants), estimated dosage varies from 2.6 to 3.9 mSv [6,7]. In a recent study it is stated that one CT scan in early age increases the risk of developing lymphoid or myeloid malignancies by about 16% [9]. The ALARA principle (As Low As Reasonably Achievable) forces clinicians to continuously evaluate imaging practices and explore alternative approaches that maintain diagnostic accuracy while prioritizing patient safety [10]. Several radiation-free imaging modalities have emerged as viable diagnostic alternatives, showing sensitivity and specificity comparable to 3DCT. Building on these advancements, our study systematically reviews the diagnostic performance, their advantages and limitations, and proposes a novel multimodal framework to enable comprehensive, radiation-free assessment of craniosynostosis—the Advanced Radiation-Free Cranial and Neural Assessment (ARCANA) protocol.

Three-dimensional stereophotogrammetry (3DSPG) provides objective, quantitative assessment of external craniofacial morphology with high accuracy and reproducibility [11,12,13,14,15,16]. High-resolution ultrasonography (US) serves as an effective, non-invasive, point-of-care tool for direct suture assessment in infants, often clarifying diagnoses without additional imaging requirements [17,18]. Most notably, advanced magnetic resonance imaging (MRI) sequences can generate high-resolution three-dimensional bone renderings that challenge computed tomography visualization while simultaneously providing superior soft tissue characterization [19,20,21].

Despite ongoing technological advances, significant global disparities persist in the availability and accessibility of advanced imaging modalities, particularly MRI and CT. These differences are especially pronounced between high-income and low-resource settings, where infrastructure, equipment, and trained personnel may be limited. Such variability poses a major challenge for the universal implementation of standardized diagnostic pathways for craniosynostosis. In this context, ultrasonography emerges as a particularly valuable tool, given its portability, absence of radiation, and substantially lower cost compared to CT and MRI—making it especially advantageous in low-income settings. Currently, no diagnostic protocol integrates the full spectrum of available modalities into a single, adaptable, and radiation-free framework. This gap in the literature underscores the need for a comprehensive and globally applicable solution—one that balances diagnostic accuracy with safety, feasibility, and resource sensitivity. The idea behind the ARCANA protocol was to address this unmet need.

The aim of this study is to synthesize the available evidence and clinical rationale concerning current diagnostic modalities in craniosynostosis, and to propose the Advanced Radiation-Free Cranial and Neural Assessment (ARCANA) Protocol as a unified, multimodal framework for its evaluation. This protocol integrates modern diagnostic modalities into a standardized assessment pathway, aiming to eliminate ionizing radiation exposure while maximizing patient safety and delivering comprehensive neurocranial insights.

## 2. Methods

This systematic review adheres to the Preferred Reporting Items for Systematic Reviews and Meta-Analyses (PRISMA) guidelines [22]. This systematic review was registered in the Open Science Framework (OSF) on 29 September 2025. The protocol, including search strategy and inclusion criteria, is accessible at https://osf.io/tng8h, accessed on 29 September 2025. A comprehensive literature search was performed using PubMed and the Cochrane Library, covering publications from 2015 to the present, restricted to English-language and full-text articles. The search string used in both databases was: “craniosynostosis” AND “computed tomography” OR “magnetic resonance imaging” OR “ultrasound” OR “ultrasonography” OR “stereophotogrammetry” OR “radiation dose”. Studies were eligible for inclusion if they evaluated these diagnostic modalities, reported diagnostic accuracy metrics, quantified radiation exposure, or assessed neurocranial outcomes in patients with craniosynostosis. All incomplete articles and those not written in English were excluded. During data extraction, additional exclusions were made for studies that did not involve humans, children, or craniosynostosis. Studies focusing on non-clinical diagnostics, prenatal evaluations, or those with experimental designs were also excluded.

The data across the included studies were too heterogeneous (in imaging techniques, diagnostic parameters, and outcome reporting) to permit valid statistical pooling or meta-analysis. Also, given the large number of included studies and the diversity of outcomes and imaging modalities, compiling all study characteristics into a single summary table was neither feasible nor informative. Accordingly, findings were organized and synthesized into separate summary tables for each diagnostic modality (3DCT, 3DSPG, US and MRI), with key qualitative findings highlighted for each method. This approach maintains PRISMA-compliant transparency while enhancing readability and clinical relevance. The review identified recurring themes, including concerns over radiation exposure, diagnostic limitations of current imaging protocols, and emerging evidence in support of radiation-free alternatives.

The methodological quality and risk of bias in diagnostic studies were assessed using the Quality Assessment of Diagnostic Accuracy Studies-2 (QUADAS-2) tool. To evaluate the certainty of evidence supporting each imaging modality, we applied the GRADE (Grading of Recommendations, Assessment, Development and Evaluations) framework, as recommended for diagnostic accuracy reviews. Five domains were assessed for each outcome and modality: (1) risk of bias (based on QUADAS-2 results), (2) inconsistency of results across studies, (3) indirectness in population or test comparisons, (4) imprecision of estimates (sample size, confidence intervals), and (5) potential publication bias. Each imaging modality (3DCT, MRI, Ultrasound, 3DSPG) was evaluated across key diagnostic outcomes: suture visualization, bone thickness and density, intracranial anatomy visualization, radiation exposure, and surgical planning capability. Certainty was rated as high, moderate or low.

This structured methodology aimed to define diagnostic requirements and identify optimal imaging modalities for each stage of patient management, culminating in the proposal of a novel, radiation-free protocol for CS evaluation.

## 3. Results

**Figure 1:**
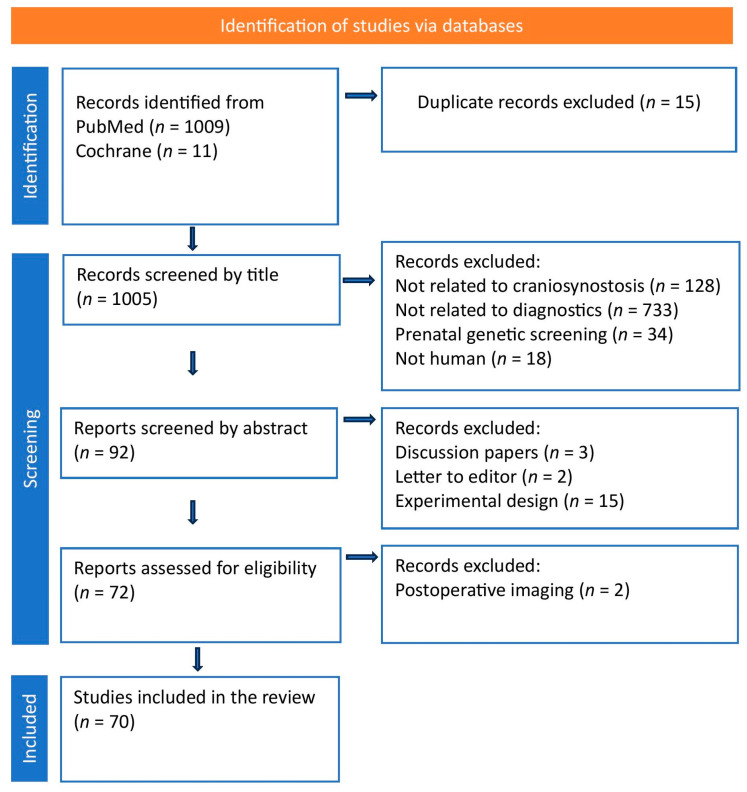
PRISMA flow diagram.

Two independent reviewers performed a systematic search of PubMed and the Cochrane Library for publications from 2015 to the present, limited to English-language, and full-text articles. The initial search identified 1020 articles. After removing 15 duplicates and screening titles and abstracts, 72 full-text articles were assessed for eligibility. Ultimately, 70 studies met inclusion criteria for systematic analysis, as shown in the PRISMA flow diagram (Figure 1). Two independent reviewers conducted all stages of the review process, including title and abstract screening, full-text eligibility assessment, and data extraction, with disagreements resolved by consensus or consultation with a third reviewer when necessary.

### 3.1. Risk of Bias Assessment

**Chart 1:**
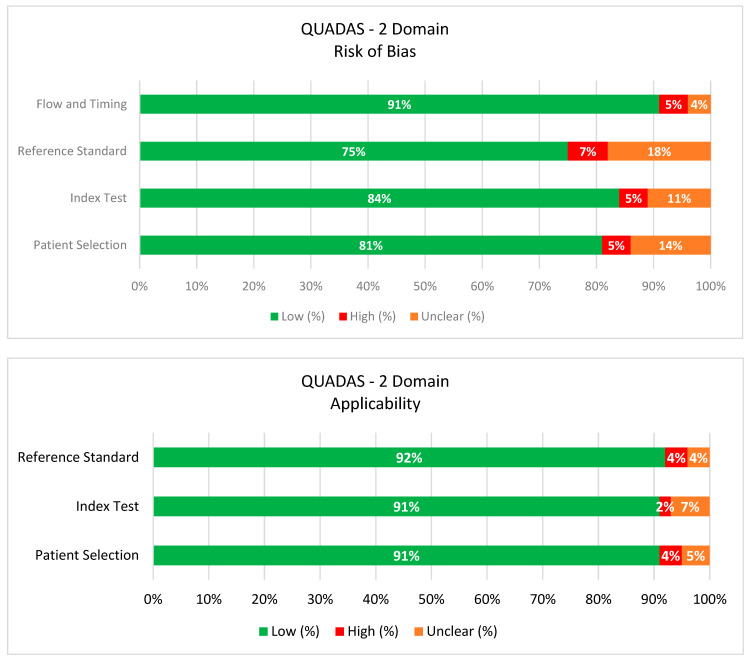
QUADAS–2 results: Proportion of studies with low, high and unclear risk for bias and applicability.

Quality appraisal was conducted for each included study using the QUADAS-2 tool, with results summarized in the Chart 1. The QUADAS-2 assessment revealed that most studies were rated as having a low risk of bias in the domains of the index test and reference standard, reflecting the use of validated imaging techniques and appropriate diagnostic confirmation. In contrast, an unclear risk of bias was frequently observed in the domains of patient selection and flow/timing. This was primarily due to retrospective study designs, insufficient reporting of inclusion criteria, and variability in the interval between imaging and reference diagnosis. A smaller number of studies showed a high risk of bias in patient selection, mainly related to non-consecutive recruitment or exclusion of complex cases. Despite these limitations, applicability concerns were generally low, indicating that the included studies are broadly representative of real-world diagnostic practice in craniosynostosis imaging.

### 3.2. GRADE Summary of Certainty

Application of the GRADE framework revealed high certainty supporting 3DCT in suture visualization, bone assessment, and surgical planning, although it ranked low for radiation safety, due to inherent ionizing exposure. MRI demonstrated moderate certainty across most outcomes, with particular strength in intracranial assessment and radiation safety. Ultrasound achieved high certainty for suture visualization in infants, but it is limited by operator dependence and inability to visualize intracranial structures if the fontanel is not open. 3DSPG received low to very low certainty for most outcomes due to its inability to visualize internal structures, despite high safety and accessibility. These findings reinforce the ARCANA protocol’s structured use of modalities: ultrasound for triage, MRI as the primary diagnostic platform, and 3DSPG for morphology tracking—offering a multimodal, radiation-free, patient-centered alternative to CT-based workflows (Table 1).

### 3.3. Three-Dimensional Computed Tomography: The Reference Standard

Craniosynostosis diagnosis traditionally relies on physical examination and craniometric assessment [18,19]. According to current guidelines, children with suspected CS first undergo skull radiographs in multiple projections [23]. If initial findings are inconclusive, repeat assessment is advised within 1–2 months. 3DCT is performed when radiographs confirm CS, fail to exclude it, or when suspicion remains high. 3DCT retains reference status due to high diagnostic reliability, fast acquisition without the need for sedation, critical role in surgical planning, and wide accessibility [24,25].

The fundamental radiobiological principle underlying pediatric imaging safety is that ionizing radiation exposure carries inherent stochastic risks, meaning that even significantly reduced doses contribute to cumulative lifetime cancer risk with no established safe threshold [5,26,27,28,29]. The developing pediatric brain, eyes, and thyroid gland demonstrate particular sensitivity to radiation effects [8]. Children with CS typically require repeated imaging for diagnosis, operative planning, and surveillance. This cumulative radiation contributes to heightened lifetime malignancy risk [8]. Due to the limited diagnostic value of routine skull radiographs in CS and the proven risk of radiation-induced intracranial tumors, best practice discourages routine X-rays in suspected cases in favor of direct referral to craniofacial specialists [30,31] (Table 2).

The growing awareness of radiation risks in pediatric populations has led to the development of low-dose and ultra-low-dose computed tomography protocols for CS evaluation [28,29,37,39,40,45]. Proponents of this approach report significant effective radiation dose reductions of 90–98% through advanced iterative reconstruction algorithms and deep learning reconstruction techniques, achieving exposure comparable to standard skull radiographs while retaining suture visualization quality [33,43].

While low-dose and ultra-low-dose protocols may provide adequate basic suture assessment, radiation dose reduction often involves compromise in image quality (particularly in the skull base region) that can impact clinical decision-making [19,46]. Study methodology, heterogeneity and reporting variability further limit generalizability of dose-reduction results [39]. Crucially, even the most advanced 3DCT technology supplies minimal detail regarding intracranial structures, cerebrospinal fluid dynamics, venous anatomy, or white matter organization [47]. 3DCT has sensitivity about 90% and specificity 90–100% in the diagnosis of closed suture [44].

### 3.4. Three-Dimensional Stereophotogrammetry

3DSPG stands as a non-invasive and radiation-free tool for capturing the external craniofacial surface anatomy (Table 3) [11,14,15,48]. This technique uses optical sensors to acquire multiple two-dimensional images from different perspectives, which are then algorithmically reconstructed into a precise, full three-dimensional digital cranial model [49]. This technology offers several clinically relevant capabilities that support its integration into CS assessment protocols:

- Objective Morphometric Assessment: 3DSPG enables quantitative analysis of cranial deformities that exceeds the limitations of subjective clinical assessment and traditional anthropometric measures [50,51]. Advanced analytic techniques, such as statistical shape modeling and principal component analysis, facilitate complex shape quantification, generate patient-specific digital models, and offer continuous severity scoring systems for CS [13,52,53].
- Longitudinal Tracking: The non-invasive nature and rapid acquisition time make 3DSPG particularly suitable for longitudinal tracking of cranial morphology and assessment of intervention efficacy over time [54,55].
- Clinical Accessibility and Patient Tolerance: 3DSPG acquisition is rapid, non-invasive, and does not require sedation, making it highly suitable for the pediatric population [56,57].
- Integration with Artificial Intelligence (AI): Machine learning models utilizing large 3DSPG datasets enable automated CS diagnosis and subtyping with reported high sensitivity and specificity in the literature [12,50,56,58,59,60].

**Table 3:** Summary of Three-dimensional Stereophotogrammetry (3DSPG) in the Diagnosis of Craniosynostosis [11,12,13,14,15,24,48,49,50,52,54,56,57,58,61,62,63,64].

3DSPG Feature	Normal Finding	Abnormal in CS	Clinical Utility	Measurement Method/Tools
**Cranial Symmetry**	Bilateral, mirrored shape of cranial vault and face	Asymmetry of skull/facial contours	Quantifies degree and type of cranial asymmetry	Superimposition, symmetry analysis software, color maps
**Suture Ridge & Depression**	Smooth bone surfaces and suture lines	Visible bony ridges or suture depressions	Direct marker of fused or compensatory bone growth	3D surface rendering, linear distance and ridge mapping
**Cranial Vault Volume/Shape**	Evenly distributed cranial volume	Regional flattening or bossing	Evaluates extent/location of vault deformity	Region-of-interest volumetry, surface deviation mapping
**Orbital/Facial Displacement**	Proportional, centered position of orbits/ears	Orbits, ears, or midface shifted/asymmetric	Detects associated orbital/ear involvement	Landmark-based measurement, 3D coordinate analysis

### 3.5. Ultrasonography

High-resolution cranial ultrasonography (US), typically performed with a high-frequency linear probe (11–18 MHz), serves as a sensitive point-of-care tool for real-time assessment of suture patency in infants (Table 4) [17,18,65]. Diagnostic capabilities include:

- **Direct Visualization:** A patent suture is visualized as a hypoechoic gap between two echogenic bony plates, whereas a fused suture is identified by the loss of this gap and the presence of a continuous echogenic bony ridge [66]. The presence of acoustic shadowing (brain shadow sign) due to sound wave obstruction at closed sutures further enhances diagnostic accuracy [67].
- **Early Detection and Screening:** US can detect suture patency prenatally as early as the first trimester. Postnatal US demonstrates high sensitivity (71–100%) and specificity (86–100%) for single-suture synostosis, especially in infants under 8 months of age [68,69].
- **Clinical Strengths:** US is portable, does not require sedation, and can be performed bedside, which minimizes stress for the child [70]. This modality proves particularly valuable for differentiating deformational plagiocephaly from true synostosis, potentially eliminating the need for additional imaging when suture patency is confirmed [71].

**Table 4:** Summary of Ultrasonographic Findings in the Diagnosis of Craniosynostosis [17,18,25,65,66,67,68,69,70,71,72].

US Feature	Normal Finding	Abnormal in CS	Clinical Utility	Imaging Plane/View or Technique
**Suture Patency**	Open, hypoechoic sutures	Absent, fused, or hyperechoic/narrowed line	Directly identifies fused sutures	Coronal or sagittal view over the suture
**Suture Morphology**	Thin, regular echogenic lines (<3 mm)	Thickened, irregular, or discontinuous suture	Suggests abnormal fusion or bone reaction	High-frequency linear probe, zoomed scan
**Bone Edges/Overriding**	Smooth, aligned cranial bones	Step-off, overriding bone at suture	Supports diagnosis, shows compensatory bone changes	Oblique sweep across suture lines
**Cranial Contour**	Regular, symmetric skull shape	Abnormal head shape (ridge, asymmetry, flattening)	Assesses severity and surgical indication	Panoramic scan or composite sweep

### 3.6. Magnetic Resonance Imaging

Recent MRI technical improvements have evolved to address prior limitations in bone visualization while maintaining superior soft tissue contrast capabilities (Table 5). Advanced MRI techniques now offer detailed assessment of both bone and neural anatomy without ionizing radiation exposure:

- Advanced Bone Imaging Capabilities: Traditional MRI limitations in cortical bone visualization stemmed from extremely low free-water content and short T2 relaxation times [73]. Novel MRI sequences have overcome this drawback through several approaches:
  ○ Zero Echo Time (ZTE) and Ultrashort Echo Time (UTE) sequences capture signals within microseconds after frequency excitation, capturing rapidly decaying signals from bound water in cortical bone [74]. These techniques enable high-contrast bone visualization producing 3DCT-comparable images of skull anatomy [19,20,21].
  ○ Golden Angle Volumetric Interpolated Breath-hold Examination (GA-VIBE) offers motion-robust cranial bone imaging with reported sensitivity of 97% and specificity of 96% for detecting suture closure compared to standard 3DCT [20].
  ○ Black Bone MRI (BBMRI) utilizes conventional sequences with parameter modifications, making it more widely available on existing MRI systems than specialized UTE/ZTE sequences [75]. This approach provides high-fidelity cranial reconstructions adequate for distinguishing craniosynostosis from positional deformities [76,77].
- Intracranial Assessment Advantages: MRI’s superior soft tissue contrast enables comprehensive evaluation of intracranial anatomy, revealing pathological details that other imaging modalities may not detect. This includes assessment of associated anomalies such as ventriculomegaly, Chiari malformation, and other developmental abnormalities commonly observed in CS [78,79,80]. These findings can significantly alter surgical planning and patient management strategies [41].
- Advanced Neuroimaging Capabilities: Diffusion Tensor Imaging (DTI) provides assessment of white matter tract integrity, organization, and development [4,47]. This MRI technique offers objective evaluation of the potential neurodevelopmental impact of cranial constraint by measuring the physical properties of neural pathways [4].
- MR Venography (MRV) and Phase-Contrast MRI enable evaluation of intracranial venous systems and cerebrospinal fluid flow dynamics [81]. These assessments provide insights into venous outflow patterns and CSF flow that may be altered in complex CS cases [82,83].
- Comprehensive Single-Session Assessment: The integration of advanced osseous imaging sequences with standard brain imaging protocols enables extensive evaluation of both skeletal and neural anatomy in a single examination session [84]. This approach updates diagnostic workflows while providing more complete anatomical information than traditional single-modality approaches [85,86]. The development of sophisticated machine learning frameworks can even synthesize high-resolution pseudo-CT images directly from MRI data, demonstrating excellent bone segmentation accuracy [87,88,89].
- Availability: Advanced MRI sequences for bone visualization (ZTE, UTE, GA-VIBE, BBMRI) are not yet widely available across all imaging centers, limiting their routine clinical implementation.

**Table 5:** Summary of Magnetic Resonance Imaging (MRI) in the Diagnosis of Craniosynostosis [3,4,19,20,21,74,75,76,77,82,84,85,86,87,88,89,90,91].

MRI Feature	UTE	ZTE	GA-VIBE	BBMRI
**Core Principle**	Captures signal from tissues with ultra-short T2WI (bone) by using echo times < 1 ms	Uses effectively zero echo time to image tissues with extremely short T2WI (bone)	3D T1-GRE with golden-angle radial sampling for improved motion robustness and bone contrast	3D T1-GRE with short TE, low flip-angle for pronounced bone/soft tissue contrast
**Bone Visualization**	Excellent; shows cortical bone, high contrast vs. soft tissue	Excellent; shows bone with high contrast, “CT-like” appearance	Excellent; BBMRI-like, strong delineation of cortical bone	Very Good; bone edges and surfaces are highlighted, though less sensitive than UTE/ZTE
**Soft Tissue Contrast**	Moderate; focus is on bone/tendons, not ideal for soft tissue	Moderate; same as UTE	Good; provides usable soft tissue images along with bone	Good; can visualize soft tissue, but main use is for bone
**3D Isotropic Imaging**	Yes, multiplanar and 3D modeling	Yes, 3D reconstructions	Yes, 3D surface renderings/virtual models	Yes, thin slices, 3D reconstructions easily obtained
**Motion** **Robustness**	Good; fast acquisition, but may still be susceptible to motion	Good; very fast acquisition, less motion sensitivity	Excellent; radial sampling is highly motion-resistant	Good; fast acquisition times reduce motion artifacts
**Availability**	Limited; needs dedicated hardware/software on MRI systems	Limited; mostly on high-end, research or new MRI systems	Variable; available on newer platforms, research, and pediatric centers	Increasing; can be implemented on many current MRI scanners
**Limitations**	Hardware/sequence availability; may produce noise if not optimized	Not widely available; complex post-processing often required	Mainly motion-resistance improvement and surface bone, less inner bone	Slightly lower bone detail vs. UTE/ZTE; parameter tuning required

## 4. Discussion

Multiple studies have demonstrated that high-quality research papers representing Level I or II evidence constitute only a minor fraction of the neurosurgical literature [92,93]. This limited availability of high-level evidence has significant implications for the strength and reliability of clinical practice guidelines and protocols in neurosurgery. Nevertheless, fundamental clinical reasoning combined with existing evidence-based principles strongly supports a transformative shift toward safer diagnostic methodologies and comprehensive evaluation protocols for craniosynostosis patients.

Across the 70 included studies, 3DCT, ultrasonography, and MRI were investigated, with 3DCT persisting as the diagnostic gold standard, despite growing support for radiation-free alternatives. However, robust evidence now supports a paradigm shift toward radiation-free, multimodal strategies that are particularly valuable in early diagnosis and preoperative planning. High-resolution cranial ultrasonography (US) demonstrates high sensitivity and specificity (86–100%) for real-time assessment of suture patency, especially in infants [68,69]. Meulstee et al. demonstrated that three-dimensional stereophotogrammetry (3DSPG) provides cranial shape measurements comparable to CT-derived models, supporting its use for postoperative monitoring [56]. Similarly, de Jong et al. confirmed that 3DSPG achieves sufficient surface accuracy to replace CT in the evaluation of nonsyndromic cases [50]. Regarding MRI, several studies reported that advanced bone-selective sequences such as ZTE/UTE and GA-VIBE yield diagnostic accuracy comparable to CT, with sensitivity and specificity values exceeding 95% for suture patency assessment [20,21,77,86]. Our systematic review critically evaluated the diagnostic accuracy, clinical applicability, and limitations of contemporary imaging modalities for craniosynostosis assessment.

The clinical emphasis on reduced dose 3DCT scanning represents a cautious modification that maintains adherence to established but suboptimal practices [29,40,43,46]. True progress in clinical medicine demands the adoption of new technologies that eliminate the inherent hazards of conventional methods while simultaneously delivering qualitatively enhanced and more thorough diagnostic data. The medical imaging field must progress beyond mere radiation dose optimization toward technologies that are inherently safe and diagnostically more informative. Accordingly, modern craniosynostosis management demands imaging efficiency grounded in patient safety, diagnostic precision, and neurodevelopmental insight. An integrated diagnostic ecosystem should leverage multiple advanced, radiation-free modalities, assigning each unique, synergistic function within a comprehensive clinical workflow.

3DSPG emerged as an efficient and cost-effective diagnostic tool. It is non-invasive, radiation-free and requires no sedation, rendering it especially suitable for pediatric patients [11,14,15,48]. Studies report a strong 3DSPG correlation with 3DCT-based severity scoring and effectiveness in monitoring treatment progress [13,16]. This capability supports evidence-based evaluation of therapeutic interventions without repeated radiation exposure. The emergence of smartphone-based solutions further enhances accessibility for routine clinical practice and telemedicine applications [61,94]. These practical advantages support implementation across diverse clinical settings. While 3DSPG primarily captures external soft tissue morphology and cannot directly visualize cranial sutures or intracranial structures, its role as a radiation-free, quantifiable assessment tool for external cranial morphology provides valuable clinical information for diagnostic and monitoring purposes.

Among the various imaging modalities evaluated in this review, US emerged as a valuable first-line tool for the visualization of suture patency in infants. Its primary limitations include operator dependence and inability to generate detailed three-dimensional reconstructions or visualize skull base anatomy. However, its role as a screening and triage tool provides valuable clinical information for guiding subsequent diagnostic decisions.

Recent advancements in MRI technology, including ZTE, UTE, GA-VIBE, and BBMRI sequences, have significantly improved the modality’s ability to visualize cranial sutures and bony anatomy with accuracy comparable to 3DCT and without ionizing radiation. In addition to bone imaging, MRI offers superior soft tissue contrast, enabling detailed assessment of intracranial structures, associated anomalies, venous outflow, CSF dynamics, and even white matter integrity. By combining diverse capabilities, MRI has the potential to become the comprehensive workhorse of CS evaluation, providing a truly holistic, radiation-free assessment of the craniofacial domain and its critical neurological implications. This multi-parametric approach moves the field far beyond simple bone visualization, leading to an era of high-accuracy neurocranial care. The limitation of MRI in the pediatric population is the need for sedation and initial high cost of the equipment.

Despite all technological advances, the adoption of new modalities into routine CS management requires careful consideration of clinical, logistical, and economic factors.

## 5. The Arcana Protocol: An Integrated Clinical Algorithm

The ARCANA Protocol (Advanced Radiation-Free Cranial and Neural Assessment) is a proposed clinical framework developed to address limitations in current CS imaging practices. The protocol development utilized a structured evidence synthesis approach combining literature review, clinical expertise, and established imaging principles.

### 5.1. Protocol Structure and Clinical Algorithm

The ARCANA Protocol proposes an integrated clinical algorithm utilizing advanced imaging modalities through a precise approach that aligns diagnostic efforts with critical therapeutic windows and surgical decision-making milestones. This systematic framework ensures that every patient receives appropriate assessment while eliminating unnecessary radiation exposure and providing superior diagnostic information compared to the traditional CT-centric approach. The protocol adapts to individual patient needs while maintaining consistent, evidence-based standards that optimize both immediate surgical outcomes and long-term neurodevelopmental trajectories.

**Figure 2:**
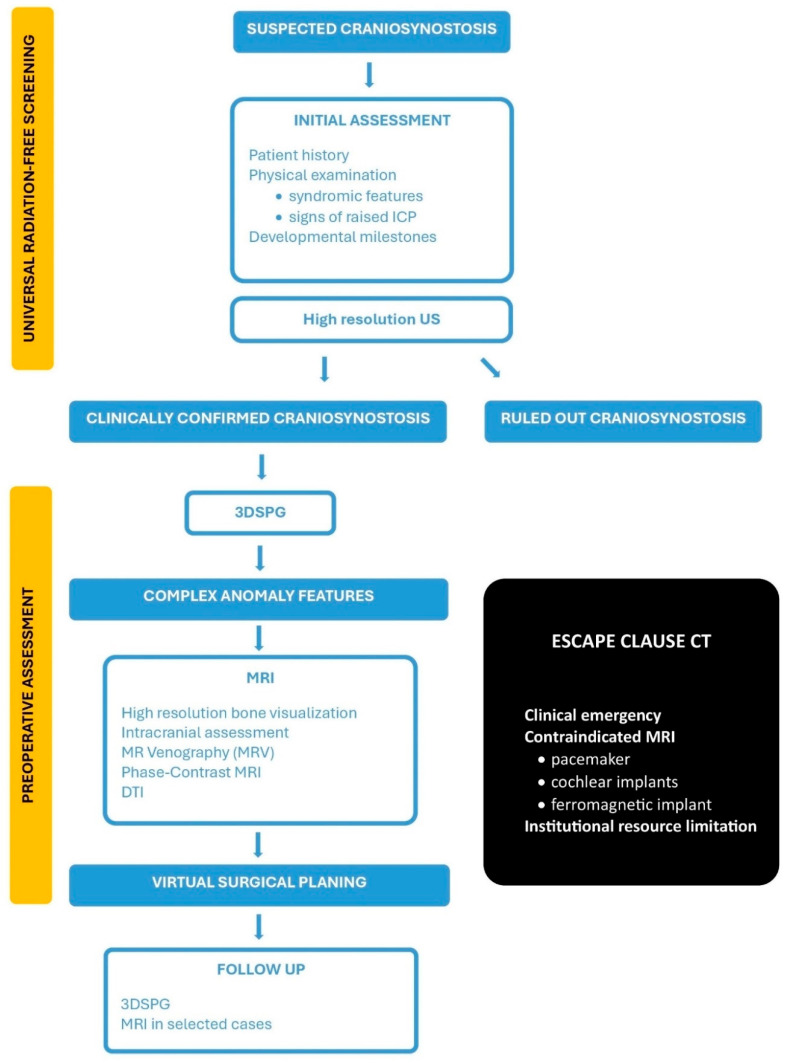
ARCANA protocol. (US—Ultrasound; 3DSPG—Three-dimensional stereophotogrammetry; MRI—Magnetic Resonance Imaging; DTI—Diffusion Tensor Imaging).

The protocol operates through a structured two-step process: universal radiation-free screening for all patients presenting with suspected CS, followed by definitive assessment for confirmed or highly suspected cases requiring surgical intervention (Figure 2).

#### 5.1.1. STEP 1: Universal Radiation-Free Screening

##### Applied to All Children with Suspected Craniosynostosis

**Initial Assessment**

Every infant or child presenting with abnormal head shape undergoes clinical examination by an experienced craniofacial specialist. Typically, a neurosurgeon, maxillofacial or plastic surgeon, depending on national practice—or by a multidisciplinary team member with extensive experience in craniosynostosis diagnosis. This initial assessment includes detailed history taking, physical examination for syndromic features, evaluation of developmental milestones, and detection of signs of elevated intracranial pressure. This clinical foundation guides the subsequent imaging pathway and establishes the diagnostic context for all subsequent evaluations.

Following clinical examination, high-resolution cranial ultrasound serves as the primary point-of-care screening modality for direct suture patency assessment.

The combination of clinical assessment and ultrasound findings leads to one of two distinct pathways:

**PATHWAY A: Conservative Management (Craniosynostosis Ruled Out)**

When cranial ultrasound unequivocally confirms patent sutures across all examined areas (metopic, bilateral coronal, sagittal and bilateral lambdoid suture), the patient enters conservative management. By definition, a suture is patent when using a linear probe, perpendicularly to the surface of the head, you can clearly visualize a continuous hypoechoic suture line [47,66]. From the suture opening, the dura can be seen as a faint echogenic line [72]. Supportive features include a head shape deformity consistent with positional molding and no clinical evidence of syndromic features or raised intracranial pressure.

**PATHWAY B: Preoperative Assessment (Craniosynostosis Confirmed)**

Patients undergo 3DSPG as the universal baseline assessment tool. This technique provides precise, quantitative documentation of external cranial morphology, enabling objective severity scoring, asymmetry quantification, and a permanent digital record for longitudinal follow-up. The non-invasive nature, rapid acquisition time, and absence of sedation requirements make this technology ideal for repeated measurements throughout the patient journey without causing distress or cumulative risk.

Patients requiring definitive assessment undergo a single, comprehensive MRI examination designed to provide complete diagnostic information for surgical planning. This multi-sequence MRI protocol systematically addresses all diagnostic requirements and may even surpass the information yield of traditional CT-based approaches. Depending on the imaging objectives and resource availability, the protocol for craniosynostosis may include only bone-focused sequences for suture assessment or additional sequences to evaluate associated brain anomalies.

High-resolution Bone Visualization can be achieved with advanced MRI sequences (ZTE/UTE, GA-VIBE, BBMRI). These techniques provide precise evaluation of suture patency, cranial vault morphology, and skull base anatomy. The resulting high-fidelity MRI data is suitable for both diagnostic interpretation and virtual surgical planning applications. ZTE and UTE MRI sequences are preferred over GA VIBE and BBMRI for imaging cranial sutures, as they provide superior bone detail and soft tissue contrast with reduced susceptibility to motion artifacts. When intracranial anatomy also needs to be evaluated, additional sequences such as T1-weighted (3D MPRAGE), T2-weighted (3D SPACE or CUBE), FLAIR (Fluid-Attenuated Inversion Recovery), and SWI (Susceptibility-Weighted Imaging) are typically included in the same examination. This comprehensive assessment reliably detects and characterizes associated intracranial pathologies commonly observed in craniosynostosis, including ventriculomegaly, Chiari malformations, and other developmental anomalies that are critical for surgical planning and overall patient management.

In selected cases, advanced neurocranial analysis can also be performed. DTI provides exceptional insight into white matter tract integrity and neural connectome organization, revealing the quantitative impact of cranial constraint on brain development. This objective assessment of neural pathway integrity offers prognostic information unavailable through traditional imaging approaches and establishes baseline measurements for long-term neurodevelopmental monitoring. MR Venography (MRV) and Phase-Contrast MRI evaluate intracranial venous anatomy and cerebrospinal fluid flow dynamics, respectively. These assessments identify venous outflow obstruction, quantify CSF flow patterns, and provide critical information regarding elevated intracranial pressure risk, all of which directly influence surgical strategy and timing decisions.

Finally, high-fidelity three-dimensional anatomical data derived entirely from MRI sequences can be seamlessly integrated into advanced Virtual Surgical Planning (VSP) platforms. This enables precise osteotomy planning, custom cutting guide design, and accurate post-operative morphology prediction, enhancing surgical precision and patient outcomes without any reliance on CT-derived data.

While these advanced MRI sequences hold promise, further validation is required before they can fully replace 3DCT, which remains essential for evaluating complex craniofacial anomalies, skull base pathology, and surgical planning. Moreover, the limited availability of these modalities across clinical centers currently restricts their integration into standardized protocols such as ARCANA, underscoring the need for broader implementation.

**Postoperative Surveillance and Long-Term Management**

Post-operative monitoring maintains the protocol’s commitment to radiation elimination through continued use of 3DSPG for objective, longitudinal tracking of external cranial morphology and surgical outcomes. This approach enables precise documentation of head shape normalization and surgical success without cumulative radiation exposure over the patient’s lifetime.

Follow-up MRI remains reserved for specific clinical indications, including syndromic patients, those with persistent neurocognitive concerns, suspected elevated intracranial pressure recurrence, or complex cases requiring detailed assessment of surgical impact on brain development. This targeted approach provides continued monitoring of brain morphology, white matter development, and objective documentation of surgical intervention effects on the developing nervous system.

The protocol’s comprehensive baseline dataset enables correlation of quantitative imaging biomarkers with long-term neurodevelopmental outcomes, establishing objective predictive models that inform prognosis and guide intervention strategies. This approach transforms CS management from reactive treatment to predictive, personalized care optimization.

#### 5.1.2. The Escape Clause: When 3DCT Is Justified?

The escape clause represents a carefully constructed safety valve within the ARCANA Protocol, designed to address genuine clinical emergencies while maintaining the overarching commitment to radiation elimination. Its existence should be interpreted as recognition that medicine occasionally presents scenarios where immediate patient safety considerations may temporarily override radiation safety principles. The application of 3DCT should be strictly reserved for rare and exceptional circumstances where MRI is either contraindicated or logistically impossible, serving as a last resort rather than a convenient alternative:

- Complex Surgical Scenarios with Diagnostic Uncertainty
  These scenarios are rare and involve patients whose comprehensive, radiation-free imaging yields inconclusive results, leading to significant diagnostic uncertainty and surgical risk. All available radiation-free alternatives must have been exhausted, and the information potentially gained from 3DCT must be both essential and unobtainable through other means.
- Absolute MRI Contraindications
  These are patients with life-threatening contraindications to MRI, such as those with incompatible implanted devices (e.g., specific pacemakers, some cochlear implants, or other ferromagnetic devices) that preclude safe MRI scanning. Such cases are exceptionally rare in pediatric craniosynostosis but require comprehensive pre-scan screening and device compatibility verification.
- Institutional Resource Limitations
  In settings where advanced MRI sequences or specialized neuroradiological expertise are genuinely unavailable, 3DCT may be needed. This situation should be viewed as a temporary institutional limitation that is not acceptable in the long-term clinical practice. Educational initiatives must be established to provide specialized training for radiologists, neurosurgeons, and technical personnel, enabling them to master advanced MRI interpretation techniques and radiation-free diagnostic approaches.

Healthcare institutions must prioritize the development of ARCANA-compatible MRI infrastructure. All uses of 3DCT under the escape clause should be systematically tracked, documenting frequency, justification, clinical outcome, and opportunities for process improvement. These data should inform ongoing efforts to eliminate radiation exposure by improving MRI capacity and refining clinical protocols. As MRI technology advances and institutional capacity increases, the frequency of justifiable 3DCT exceptions should steadily decline toward zero.

#### 5.1.3. Future Horizons for the Proposed ARCANA Protocol

The proposed ARCANA Protocol is a carefully developed framework aimed at transforming CS imaging and establishing an international standard of care. Its development followed a rigorous, multi-phase methodology. The first phase synthesized evidence from the last decade’s literature, revealing developments in imaging and exposing critical shortcomings in existing workflows, such as preventable radiation exposure, incomplete neurocranial evaluation, and a lack of objective quantitative metrics. Consultation with a broad panel of international experts would further refine clinical priorities and strengthen the foundation of the proposed diagnostic algorithm.

The subsequent phases of protocol development focus on thorough validation, strategic implementation, and global spreading to ensure global clinical impact. This comprehensive approach should include the design and execution of prospective, multi-center clinical trials to confirm the ARCANA Protocol’s diagnostic accuracy, surgical planning efficacy, and long-term influence on neurodevelopmental outcomes compared to current standards. Formal endorsement from relevant professional societies should be pursued to incorporate ARCANA into clinical guidelines, culminating in widely cited publications that establish its global authority.

The ARCANA Protocol’s strength is not the creation of new technology but the strategic integration and standardization of radiation-free tools into a unified, neuro-centric diagnostic process that clearly outperforms 3DCT-based standards. This proposed protocol is architected for continuous improvement, integrating advanced artificial intelligence and machine learning capabilities for automated analysis, predictive modeling, and personalized surgical planning, ensuring its ongoing evolution as the definitive standard for precision neurocranial care. This marks a fundamental recalibration of imaging practices, motivated by scientific progress and ethical imperatives.

The global adoption of the ARCANA Protocol requires a pragmatic, stepwise implementation model that enables gradual advancement toward radiation-free CS management while accommodating varying institutional capacities and resource constraints. In the initial phase, following the acquisition of high-resolution ultrasound and 3DSPG systems, institutions would require comprehensive staff training programs. During this transitional phase, as clinical expertise and confidence with the new modalities increase, high-resolution ultrasound and 3DSPG would be employed alongside CT, with a gradual reduction in CT use over time. This phased integration strategy fosters sustainable shifts in clinical practice by facilitating progressive skill acquisition, infrastructure readiness, and cultural adaptation within healthcare systems.

A significant barrier remains the higher initial acquisition cost of technology such as 3DSPG and advanced MRI. US for CS evaluation offers a low-cost imaging option, with per-scan costs significantly lower than 3DCT or MRI [18,19,68]. Expenses for 3DSPG systems are driven by cameras, lighting, computing hardware, and software, with variation according to system complexity and post-processing requirements [11]. MRI scans are far more expensive than 3DCT, and prolonged scan times usually necessitate sedation in pediatric patients to minimize motion artifacts [2]. However, these costs must be evaluated against several crucial factors. The long-term societal and individual costs associated with cumulative radiation exposure, including potential cancer surveillance, treatment, and lifelong health implications, likely outweigh the upfront investment in multimodal, comprehensive, radiation-free CS evaluation [8,27]. A recent systematic review by Colletti et al. underscores growing concern that multiple exposures to general anesthesia in children under four years of age may be associated with an increased risk of neurodevelopmental impairment, with the majority of controlled studies demonstrating significant cognitive or behavioral deficits compared to unexposed cohorts [95]. The risk of neurodevelopmental impairment appears to be modulated not only by the timing of exposure but also by the number and duration of anesthetic events, with multiple or prolonged exposures in early childhood consistently associated with adverse cognitive and behavioral outcomes across several systematic reviews [96,97]. While the need for sedation or anesthesia for young children during MRI represents a valid concern, this challenge is being actively mitigated through development of ultra-fast sequences, advanced motion-correction algorithms, and refinement of non-sedated techniques, which are continuously reducing scan times and anesthesia requirements [86,90,98]. Moreover, as outlined in the ARCANA protocol, comprehensive MRI evaluation is not intended for routine use but rather serves as a targeted adjunct in selected cases. The use of advanced bone-visualizing sequences with short acquisition times can be performed without anesthesia, similar to CT. This significantly limits the likelihood of repeated anesthesia exposures, which are a principal concern in relation to potential cognitive and behavioral sequelae in early childhood.

Another barrier is the substantial learning curve for radiologists, neurosurgeons, and craniofacial teams. Interpreting sophisticated MRI sequences and integrating its rich dataset into virtual surgical planning demands specialized expertise and dedicated training [47,75]. This will require purposeful investment in equipment and software, as well as fostering interdisciplinary collaboration across clinical and technical specialties. Nevertheless, the long-term improvements in diagnostic accuracy, surgical precision, and patient outcomes clearly justify this initial commitment.

The integration of AI and machine learning with ARCANA’s rich quantitative dataset offers significant potential to expand CS care. AI models trained on MRI and 3DSPG data can automate morphometric analysis, quantify brain and skull volumes, identify deviations from normative growth trajectories, and deliver objective, reproducible severity scores that surpass traditional measures [43,62,99]. Automation may also enable efficient, high-accuracy diagnostic tools capable of classifying CS subtypes and accelerating screening [50,58,60]. Additionally, advanced algorithms can process DTI for robust, reproducible assessment of white matter quality, uncovering abnormalities or developmental deviations not evident to the human observer [4]. AI models can predict neurodevelopmental outcomes by correlating comprehensive quantitative imaging biomarkers, including brain volume changes, DTI metrics, and cerebrospinal fluid dynamics, with long-term neurocognitive and developmental assessments, potentially guiding early, targeted interventions [63]. These tools could personalize surgical plans using precise MRI-derived morphology, patient genetics, and clinical variables, potentially forecasting disease severity and guiding individualized treatment to optimize outcomes [81,100]. Further, advances in augmented and mixed reality, powered by ARCANA’s high-resolution 3D datasets, could revolutionize surgical planning and intraoperative guidance [101,102]. Holographic skull models and overlaid anatomical data, interactively manipulated by surgeons, could enhance spatial awareness and precision beyond what traditional virtual surgical planning offers [103]. This technological integration is the next frontier, enabling the transition of CS care from reactive intervention to predictive, personalized medicine focused on both immediate and lifelong patient outcomes.

#### 5.1.4. Limitations of the Study and ARCANA Protocol

This systematic review acknowledges several important limitations that must be considered when interpreting the findings and implementing the proposed ARCANA Protocol. Although individual study quality was rigorously evaluated using the QUADAS-2 assessment tool, the substantial heterogeneity in study designs, methodologies, and outcome reporting precluded formal statistical evaluation of publication bias. Consequently, the potential influence of publication bias and selective outcome reporting cannot be definitively excluded and may impact the overall conclusions drawn from this analysis.

While the proposed protocol demonstrates significant promise as a comprehensive radiation-free imaging approach, its widespread clinical adoption requires additional validation across diverse healthcare environments. A critical limitation is the absence of formal economic analysis comparing the cost-effectiveness of the proposed protocol against conventional CT-based diagnostic pathways. An economic evaluation lies beyond the scope of the data available to the authors, particularly given the lack of country-specific information. However, the economic aspect, especially the cost–benefit of this protocol, becomes crucial when considering implementation in resource-constrained healthcare settings, where financial considerations often determine the feasibility of adopting new diagnostic technologies and protocols.

Finally, the most significant limitation remains the need for robust prospective clinical validation studies. Such validation studies are essential to establish the protocol’s clinical efficacy, safety profile, and real-world utility before recommending widespread implementation in clinical practice.

## 6. Conclusions

The management of craniosynostosis is currently experiencing a revolution, transitioning from a traditional anatomical discipline focused primarily on bone morphology to a comprehensive, data-driven, neurocentric science that integrates multiple dimensions of patient assessment. This transformation embodies a fundamental reconceptualization of craniosynostosis as a complex neurodevelopmental condition requiring sophisticated analytical approaches that extend far beyond simple bone visualization. The neurosurgical and neuroradiological communities stand at a crossroads where we can either continue accepting the limitations and risks of current practice, or embrace a transformative approach prioritizing patient safety while providing superior diagnostic information. The scientific evidence supporting this paradigm shift is clear, the technology is mostly available, and the ethical imperative is undeniable.

The proposed ARCANA Protocol represents not merely an advancement in craniosynostosis care but expresses our profession’s commitment to continuous improvement, patient safety, and evidence-based practice. The time has arrived to unequivocally accept this paradigm shift, recognizing that our patients deserve nothing less than the safest, most comprehensive diagnostic approach available. The future of patient care lies in radiation-free, brain-focused, and integrated approaches, where innovative protocols offer the strategic framework to realize this vision, and the professional responsibility for their implementation rests upon every clinician treating these at-risk pediatric patients.

## Figures and Tables

**Table 1:** GRADE summary table.

Outcome	Modality	Certainty of Evidence	Risk of Bias	Inconsistency	Indirectness	Imprecision	Publication Bias	Comments
**Suture Visualization**	3DCT	High	Low	Low	Low	Low	Low	Highly accurate, widely used as gold standard
	MRI	Moderate	Low	Moderate	Low	Moderate	Low	Advanced sequences show promising accuracy; availability varies
	US	High	Moderate	Moderate	Low	Moderate	Moderate	High sensitivity in infants
	3DSPG	Low	High	High	Low	High	Moderate	Only assesses external morphology; cannot visualize sutures
**Bone Thickness and Density**	3DCT	High	Low	Low	Low	Low	Low	High-resolution assessment of cortical bone with quantitative tools
	MRI	Moderate	Moderate	Moderate	Low	Moderate	Moderate	Emerging sequences effective, but less validated
	US	Low	High	High	High	High	High	Cannot quantify bone density
	3DSPG	Low	High	High	High	High	High	Shape surface imaging only
**Intracranial Anatomy Visualization**	3DCT	Moderate	Moderate	Moderate	Low	Low	Low	Limited soft tissue visualization
	MRI	High	Low	Low	Low	Low	Low	Excellent detailed visualization of brain, CSF, venous structures
	US	Moderate	High	High	Low	High	Moderate	Limited brain visualization, only while the fontanel is opened; not suitable for deep structures, close to the skull base
	3DSPG	Low	High	High	High	High	High	No intracranial data available
**Radiation Safety**	3DCT	Low	Low	Low	Low	Low	Low	Highest ionizing radiation burden; concern in pediatrics
	MRI	High	Low	Low	Low	Low	Low	No radiation
	US	High	Low	Low	Low	Low	Low	No radiation; easy repeated use
	3DSPG	High	Low	Low	Low	Low	Low	No radiation; rapid acquisition
**Surgical Planning Capability**	3DCT	High	Low	Low	Low	Low	Low	Detailed bone 3D modeling
	MRI	Moderate	Moderate	Moderate	Low	Moderate	Low	MRI-based VSP emerging
	Ultrasound	Low	High	High	Low	Moderate	Moderate	Very limited surgical usefulness
	3DSPG	Low	High	High	Low	Moderate	Moderate	Useful for pre/post-op morphology tracking only

**Table 2:** Summary of Three-dimensional computed tomography (3DCT) in the Diagnosis of Craniosynostosis [5,6,7,8,9,23,26,27,28,29,30,32,33,34,35,36,37,38,39,40,41,42,43,44].

3DCT Feature	Normal Finding	Abnormal in CS	Clinical Utility	Acquisition/Interpretation Method
**Suture Visualization**	Patent cranial sutures, clear suture lines	Suture fusion, absent suture, bony ridge formation	Direct identification of fused sutures	Multiplanar/3D volume rendering
**Skull Morphology**	Smooth cranial contours and adequate volume	Regional flattening, bossing, abnormal cranial shape	Objective measurement of cranial deformity	High-resolution 3D surface model reconstruction
**Bone Thickness and Density**	Normal cortical thickness and uniform bone density	Areas of thickening, abnormal density, suture overlap	Evaluation of structural changes associated with craniosynostosis	Hounsfield unit analysis, bone window adjustment
**Surgical Planning Capability**	Not applicable	Detailed mapping for osteotomies, bone defect visualization	Preoperative planning, intraoperative navigation	3DCT models with/without surgical simulation software
**Intracranial Visualization**	Mainly osseous anatomy, basic brain anatomy	Indirect findings, basic brain anatomy	Limited visualization of brain morphology and dynamics	Not primary modality for soft-tissue/brain analysis

## Data Availability

The original contributions presented in this study are included in the article/Supplementary Materials. Further inquiries can be directed to the corresponding author.

## Supplementary Materials

The following supporting information can be downloaded at: https://www.mdpi.com/article/10.3390/diagnostics15202632/s1, File S1: PRISMA 2020 for Abstracts Checklist [22]; File S2: PRISMA 2020 Checklist [22].

## References

[1] Lattanzi W., Barba M., Di Pietro L., Boyadjiev S.A. (2017). Genetic advances in craniosynostosis. Am. J. Med. Genet. Part A.

[2] Massimi L., Bianchi F., Frassanito P., Calandrelli R., Tamburrini G., Caldarelli M. (2019). Imaging in craniosynostosis: When and what?. Child’s Nerv. Syst..

[3] Brooks E.D., Yang J., Beckett J.S., Lacadie C., Scheinost D., Persing S., Zellner E.G., Oosting D., Keifer C., Friedman H.E. (2016). Normalization of brain morphology after surgery in sagittal craniosynostosis. J. Neurosurg. Pediatr..

[4] Moscarelli J., Almeida M.N., Lacadie C., Hu K.G., Ihnat J.M.H., Parikh N., Persing J.A., Alperovich M. (2024). A diffusion tensor imaging comparison of white matter development in nonsyndromic craniosynostosis to neurotypical infants. Child’s Nerv. Syst..

[5] Brenner D.J., Elliston C.D., Hall E.J., Berdon W.E. (2001). Estimated Risks of Radiation-Induced Fatal Cancer from Pediatric CT. Am. J. Roentgenol..

[6] Kharbanda A.B., Krause E., Lu Y., Blumberg K. (2015). Analysis of radiation dose to pediatric patients during computed tomography examinations. Acad. Emerg. Med..

[7] Obara H., Takahashi M., Kudou K., Mariya Y., Takai Y., Kashiwakura I. (2017). Estimation of effective doses in pediatric X-ray computed tomography examination. Exp. Ther. Med..

[8] Pearce M.S., Salotti J.A., Little M.P., McHugh K., Lee C., Kim K.P., Howe N.L., Ronckers C.M., Rajaraman P., Craft A.W. (2012). Radiation exposure from CT scans in childhood and subsequent risk of leukaemia and brain tumours: A retrospective cohort study. Lancet.

[9] Bosch de Basea M., Thierry-Chef I., Harbron R., Hauptmann M., Byrnes G., Bernier M.O., Le Cornet L., Dabin J., Ferro G., Istad T.S. (2023). Risk of hematological malignancies from CT radiation exposure in children, adolescents and young adults. Nat. Med..

[10] Yeung A.W.K. (2019). The “As Low As Reasonably Achievable” (ALARA) principle: A brief historical overview and a bibliometric analysis of the most cited publications. Radioprotection.

[11] Duncan C., Pears N.E., Dai H., Smith W.A.P., O’Higgins P. (2022). Applications of 3D Photography in Craniofacial Surgery. J. Pediatr. Neurosci..

[12] Görg C., Elkhill C., Chaij J., Royalty K., Nguyen P.D., French B., Cruz-Guerrero I.A., Porras A.R. (2024). SHAPE: A visual computing pipeline for interactive landmarking of 3D photograms and patient reporting for assessing craniosynostosis. Comput. Graph..

[13] Ho O.A., Saber N., Stephens D., Clausen A., Drake J., Forrest C., Phillips J. (2017). Comparing the Use of 3D Photogrammetry and Computed Tomography in Assessing the Severity of Single-Suture Nonsyndromic Craniosynostosis. Plast. Surg..

[14] Mertens C., Wessel E., Berger M., Ristow O., Hoffmann J., Kansy K., Freudlsperger C., Bächli H., Engel M. (2017). The value of three-dimensional photogrammetry in isolated sagittal synostosis: Impact of age and surgical technique on intracranial volume and cephalic index—a retrospective cohort study. J. Cranio-Maxillofac. Surg..

[15] Rückschloß T., Zittel S., Hassanein E., El Damaty A., Krieg S.M., Ristow O., Hoffmann J., Engel M. (2025). Photogrammetric evaluation of extended midline strip craniectomy with bilateral parietal osteotomies on frontal morphology in patients with isolated sagittal synostosis. Neurosurg. Focus..

[16] Zhao Z., Xie L., Cao D., Izadikhah I., Gao P., Zhao Y., Yan B. (2021). Accuracy of three-dimensional photogrammetry and cone beam computed tomography based on linear measurements in patients with facial deformities. Dentomaxillofacial Radiol..

[17] Pogliani L.M., Zuccotti G.V., Reggiori M., Erbetta A., Lacerenza M., Prada F., Furlanetto M., Vetrano I.G., Valentini L.G. (2022). Surface Cranial Ultrasound: The Natural Heir to X-Ray for the Screening of Skull Deformities in Infants. Ultraschall Der Med. Eur. J. Ultrasound.

[18] Rozovsky K., Udjus K., Wilson N., Barrowman N.J., Simanovsky N., Miller E. (2016). Cranial Ultrasound as a First-Line Imaging Examination for Craniosynostosis. Pediatrics.

[19] Kamona N., Jones B.C., Lee H., Song H.K., Rajapakse C.S., Wagner C.S., Bartlett S.P., Wehrli F.W. (2023). Cranial bone imaging using ultrashort echo-time bone-selective MRI as an alternative to gradient-echo based “black-bone” techniques. Magn. Reson. Mater. Phys. Biol. Med..

[20] Patel K.B., Eldeniz C., Skolnick G.B., Jammalamadaka U., Commean P.K., Goyal M.S., Smyth M.D., An H. (2020). 3D pediatric cranial bone imaging using high-resolution MRI for visualizing cranial sutures: A pilot study. J. Neurosurg. Pediatr..

[21] Saarikko A., Mellanen E., Kuusela L., Leikola J., Karppinen A., Autti T., Virtanen P., Brandstack N. (2020). Comparison of Black Bone MRI and 3D-CT in the preoperative evaluation of patients with craniosynostosis. J. Plast. Reconstr. Aesthetic Surg..

[22] Page M.J., McKenzie J.E., Bossuyt P.M., Boutron I., Hoffmann T.C., Mulrow C.D., Shamseer L., Tetzlaff J.M., Akl E.A., Brennan S.E. (2021). The PRISMA 2020 statement: An updated guideline for reporting systematic reviews. BMJ.

[23] Mathijssen I.M.J. (2021). Updated Guideline on Treatment and Management of Craniosynostosis. J. Craniofacial Surg..

[24] Bhalodia R., Dvoracek L.A., Ayyash A.M., Kavan L., Whitaker R., Goldstein J.A. (2020). Quantifying the Severity of Metopic Craniosynostosis: A Pilot Study Application of Machine Learning in Craniofacial Surgery. J. Craniofacial Surg..

[25] Kronig S.A.J., Kronig O.D.M., Vrooman H.A., Veenland J.F., Van Adrichem L.N.A. (2020). New diagnostic approach of the different types of isolated craniosynostosis. Eur. J. Pediatr..

[26] Fahradyan A., Daneshgaran G., Hoffman T.L., Wexler A., Francis S.H. (2021). Challenging the Norm: Is Routine Use of Cranial CT in Evaluation of Craniosynostosis Necessary?. J. Craniofacial Surg..

[27] Meulepas J.M., Ronckers C.M., Smets A.M.J.B., Nievelstein R.A.J., Gradowska P., Lee C., Jahnen A., van Straten M., de Wit M.-C.Y., Zonnenberg B. (2019). Radiation Exposure From Pediatric CT Scans and Subsequent Cancer Risk in The Netherlands. JNCI J. Natl. Cancer Inst..

[28] Nakai Y., Miyazaki O., Kitamura M., Imai R., Okamoto R., Tsutsumi Y., Miyasaka M., Ogiwara H., Miura H., Yamada K. (2023). Evaluation of radiation dose reduction in head CT using the half-dose method. Jpn. J. Radiol..

[29] Zarella C., Didier R., Bergquist C., Bardo D.M.E., Selden N.R., Kuang A.A. (2016). A Reduction in Radiation Exposure During Pediatric Craniofacial Computed Tomography. J. Craniofacial Surg..

[30] Dindaroglu F., Yetkiner E. (2016). Cone Beam Computed Tomography in Orthodontics. Turk. J. Orthod..

[31] O’Sullivan H., Bracken S., Doyle J., Twomey E., Murray D.J., Kyne L. (2020). X-rays had little value in diagnosing children’s abnormal skull shapes, and primary care clinicians should refer concerns to specialist teams. Acta Paediatr..

[32] Alnaif N., Zhou M., Galli R., Azzi A.J., Alamri A., Gilardino M. (2019). The Role of Preoperative Computed Tomography in Nonsyndromic Craniosynostosis. J. Craniofacial Surg..

[33] Barreto I.L., Tuna I.S., Rajderkar D.A., Ching J.A., Governale L.S. (2021). Pediatric craniosynostosis computed tomography: An institutional experience in reducing radiation dose while maintaining diagnostic image quality. Pediatr. Radiol..

[34] Bruce M.K., Mittal A.M., Whitt D.S., Flom L.L., Pfaff M.J., Losee J.E., Goldstein J.A. (2021). Computed tomography associated radiation exposure in children with craniosynostosis. Child’s Nerv. Syst..

[35] da Silva Freitas R., de Freitas Azzolini T., Shin J.H., Persing J.A. (2010). Associated (Parallel) Tomographic Findings in Patients With Single-Sutural Synostosis. J. Craniofacial Surg..

[36] Di Rocco F., Garcia-Gonzalez O., Szathmari A., Chauvel-Picard J., Beuriat P.A., Paulus C., Gleizal A., Mottolese C. (2021). Emissary veins and pericerebral cerebrospinal fluid in trigonocephaly: Do they define a specific subtype?. Child’s Nerv. Syst..

[37] Ernst C.W., Hulstaert T.L., Belsack D., Buls N., Van Gompel G., Nieboer K.H., Buyl R., Verhelle F., De Maeseneer M., de Mey J. (2015). Dedicated sub 0.1 mSv 3DCT using MBIR in children with suspected craniosynostosis: Quality assessment. Eur. Radiol..

[38] Fearon J.A., Singh D.J., Beals S.P., Yu J.C. (2007). The Diagnosis and Treatment of Single-Sutural Synostoses: Are Computed Tomographic Scans Necessary?. Plast. Reconstr. Surg..

[39] He K., Boukind A., Sanka A.S., Ribaudo J.G., Chryssofos S., Skolnick G.B., Yaeger L.B., Thomas A.M., Mian A.Y., Patel K.B. (2025). Systematic Review and Meta-Analysis of Radiation Dose Reduction Studies in Pediatric Head CT. Am. J. Neuroradiol..

[40] Montoya J.C., Eckel L.J., DeLone D.R., Kotsenas A.L., Diehn F.E., Yu L., Bartley A.C., Carter R.E., McCollough C.H., Fletcher J.G. (2017). Low-Dose CT for Craniosynostosis: Preserving Diagnostic Benefit with Substantial Radiation Dose Reduction. Am. J. Neuroradiol..

[41] Ravindra V.M., Awad A.-W., Baker C.M., Lee A., Anderson R.C.E., Gociman B., Patel K.B., Smyth M.D., Birgfeld C., Pollack I.F. (2021). Preoperative imaging patterns and intracranial findings in single-suture craniosynostosis: A study from the Synostosis Research Group. J. Neurosurg. Pediatr..

[42] Sheppard J.P., Nguyen T., Alkhalid Y., Beckett J.S., Salamon N., Yang I. (2018). Risk of Brain Tumor Induction from Pediatric Head CT Procedures: A Systematic Literature Review. Brain Tumor Res. Treat..

[43] Tao W., Goetti R. (2024). Evaluation of ultra-low-dose CT with tin filter for craniosynostosis. J. Med. Imaging Radiat. Oncol..

[44] Vannier M.W., Hildebolt C.F., Marsh J.L., Pilgram T.K., McAlister W.H., Shackelford G.D., Offutt C.J., Knapp R.H. (1989). Craniosynostosis: Diagnostic value of three-dimensional CT reconstruction. Radiology.

[45] Lyoo Y., Choi Y.H., Lee S.B., Lee S., Cho Y.J., Shin S.-M., Phi J.H., Kim S.K., Cheon J.-E. (2023). Ultra-low-dose computed tomography with deep learning reconstruction for craniosynostosis at radiation doses comparable to skull radiographs: A pilot study. Pediatr. Radiol..

[46] Uffmann M., Schaefer-Prokop C. (2009). Digital radiography: The balance between image quality and required radiation dose. Eur. J. Radiol..

[47] Russo C., Aliberti F., Ferrara U.P., Russo C., De Gennaro D.V., Cristofano A., Nastro A., Cicala D., Spennato P., Quarantelli M. (2024). Neuroimaging in Nonsyndromic Craniosynostosis: Key Concepts to Unlock Innovation. Diagnostics.

[48] Abdel-Alim T., Kurniawan M., Mathijssen I., Dremmen M., Dirven C., Niessen W., Roshchupkin G., van Veelen M.-L. (2023). Sagittal Craniosynostosis: Comparing Surgical Techniques Using 3D Photogrammetry. Plast. Reconstr. Surg..

[49] Abdel-Alim T., Iping R., Wolvius E.B., Mathijssen I.M.J., Dirven C.M.F., Niessen W.J., van Veelen M.-L.C., Roshchupkin G.V. (2021). Three-Dimensional Stereophotogrammetry in the Evaluation of Craniosynostosis: Current and Potential Use Cases. J. Craniofacial Surg..

[50] de Jong G., Bijlsma E., Meulstee J., Wennen M., van Lindert E., Maal T., Aquarius R., Delye H. (2020). Combining deep learning with 3D stereophotogrammetry for craniosynostosis diagnosis. Sci. Rep..

[51] Ramsey J.A., Stevens P.M., Coats B., Dixon T.J., Chaker S.C., Bonfield C.M., Golinko M.S. (2025). Comprehensive craniometry for sagittal synostosis. Neurosurg. Focus..

[52] Bruce M.K., Tao W., Beiriger J., Christensen C., Pfaff M.J., Whitaker R., Goldstein J.A. (2022). 3D Photography to Quantify the Severity of Metopic Craniosynostosis. Cleft Palate Craniofacial J..

[53] Tao W., Somorin T.J., Kueper J., Dixon A., Kass N., Khan N., Iyer K., Wagoner J., Rogers A., Whitaker R. (2025). Quantifying Sagittal Craniosynostosis Severity: A Machine Learning Approach With CranioRate. Cleft Palate Craniofacial J..

[54] Abdel-Alim T., Tapia Chaca F., Mathijssen I.M.J., Dirven C.M.F., Niessen W.J., Wolvius E.B., van Veelen M.L.C., Roshchupkin G.V. (2024). Quantifying dysmorphologies of the neurocranium using artificial neural networks. J. Anat..

[55] Porras A.R., Tu L., Tsering D., Mantilla E., Oh A., Enquobahrie A., Keating R., Rogers G.F., Linguraru M.G. (2019). Quantification of Head Shape from Three-Dimensional Photography for Presurgical and Postsurgical Evaluation of Craniosynostosis. Plast. Reconstr. Surg..

[56] Meulstee J.W., Verhamme L.M., Borstlap W.A., Van der Heijden F., De Jong G.A., Xi T., Bergé S.J., Delye H., Maal T.J.J. (2017). A new method for three-dimensional evaluation of the cranial shape and the automatic identification of craniosynostosis using 3D stereophotogrammetry. Int. J. Oral. Maxillofac. Surg..

[57] Petrides G., Clark J.R., Low H., Lovell N., Eviston T.J. (2021). Three-dimensional scanners for soft-tissue facial assessment in clinical practice. J. Plast. Reconstr. Aesthetic Surg..

[58] Azimi N., Talebi Rafsanjan K., Khanmohammadi Khorami M.M., Ebadifar A., Azadi A. (2025). Applications of Machine Learning in Image Analysis to Identify Craniosynostosis: A Systematic Review and Meta-Analysis. Orthod. Craniofacial Res..

[59] Elkhill C., Liu J., Linguraru M.G., LeBeau S., Khechoyan D., French B., Porras A.R. (2023). Geometric learning and statistical modeling for surgical outcomes evaluation in craniosynostosis using 3D photogrammetry. Comput. Methods Programs Biomed..

[60] Kuehle R., Ringwald F., Bouffleur F., Hagen N., Schaufelberger M., Nahm W., Hoffmann J., Freudlsperger C., Engel M., Eisenmann U. (2023). The Use of Artificial Intelligence for the Classification of Craniofacial Deformities. J. Clin. Med..

[61] Barbero-García I., Lerma J.L., Marqués-Mateu Á., Miranda P. (2017). Low-Cost Smartphone-Based Photogrammetry for the Analysis of Cranial Deformation in Infants. World Neurosurg..

[62] Beiriger J.W., Tao W., Bruce M.K., Anstadt E., Christensen C., Smetona J., Whitaker R., Goldstein J.A. (2023). CranioRate: An Image-Based, Deep-Phenotyping Analysis Toolset and Online Clinician Interface for Metopic Craniosynostosis. Plast. Reconstr. Surg..

[63] Harrison L.M., Edison R.L., Hallac R.R. (2025). Artificial Intelligence Applications in Pediatric Craniofacial Surgery. Diagnostics.

[64] Toma A.M., Zhurov A., Playle R., Ong E., Richmond S. (2009). Reproducibility of facial soft tissue landmarks on 3D laser-scanned facial images. Orthod. Craniofacial Res..

[65] Proisy M., Bruneau B., Riffaud L. (2019). How ultrasonography can contribute to diagnosis of craniosynostosis. Neurochirurgie.

[66] Pogliani L., Zuccotti G.V., Furlanetto M., Giudici V., Erbetta A., Chiapparini L., Valentini L. (2017). Cranial ultrasound is a reliable first step imaging in children with suspected craniosynostosis. Child’s Nerv. Syst..

[67] Krajden Haratz K., Leibovitz Z., Svirsky R., Drummond C.L., Lev D., Gindes L., Lerman-Sagie T., Malinger G. (2016). The ‘Brain Shadowing Sign’: A Novel Marker of Fetal Craniosynostosis. Fetal Diagn. Ther..

[68] Hall K.M., Besachio D.A., Moore M.D., Mora A.J., Carter W.R. (2017). Effectiveness of screening for craniosynostosis with ultrasound: A retrospective review. Pediatr. Radiol..

[69] Whittall I., Lambert W.A., Moote D.J., Bookland M.J., Martin J.E., Hughes C.D., Hersh D.S. (2021). Postnatal diagnosis of single-suture craniosynostosis with cranial ultrasound: A systematic review. Child’s Nerv. Syst..

[70] Proisy M., Riffaud L., Chouklati K., Tréguier C., Bruneau B. (2017). Ultrasonography for the diagnosis of craniosynostosis. Eur. J. Radiol..

[71] Marino S., Ruggieri M., Marino L., Falsaperla R. (2021). Sutures ultrasound: Useful diagnostic screening for posterior plagiocephaly. Child’s Nerv. Syst..

[72] Soboleski D., Mussari B., McCloskey D., Sauerbrei E., Espinosa F., Fletcher A. (1998). High-resolution sonography of the abnormal cranial suture. Pediatr. Radiol..

[73] More S.S., Zhang X. (2024). Ultrashort Echo Time and Zero Echo Time MRI and Their Applications at High Magnetic Fields: A Literature Survey. Investig. Magn. Reson. Imaging.

[74] Kobayashi N., Bambach S., Ho M.-L. (2021). Ultrashort Echo-Time MR Imaging of the Pediatric Head and Neck. Magn. Reson. Imaging Clin. North. Am..

[75] Ganau M., Syrmos N.C., Magdum S.A. (2022). Imaging in Craniofacial Disorders With Special Emphasis on Gradient Echo Black-Bone and Zero Time Echo MRI Sequences. J. Pediatr. Neurosci..

[76] Kuusela L., Hukki A., Brandstack N., Autti T., Leikola J., Saarikko A. (2018). Use of black-bone MRI in the diagnosis of the patients with posterior plagiocephaly. Child’s Nerv. Syst..

[77] Valeggia S., Dremmen M.H.G., Mathijssen I.M.J., Gaillard L., Manara R., Ceccato R., van Hattem M., Gahrmann R. (2024). Black Bone MRI vs. CT in temporal bone assessment in craniosynostosis: A radiation-free alternative. Neuroradiology.

[78] Cuthbert H., Gallo P., Galloway L., Goel A., Afshari F.T., Solanki G.A., Rodrigues D., Gagen R., Pepper J. (2025). Occult craniosynostosis in normocephalic children with Chiari I malformation. J. Neuroradiol..

[79] Strahle J., Muraszko K.M., Buchman S.R., Kapurch J., Garton H.J.L., Maher C.O. (2011). Chiari malformation associated with craniosynostosis. Neurosurg. Focus..

[80] Zavala C.A., Zima L.A., Greives M.R., Fletcher S.A., Shah M.N., Miller B.A., Sandberg D.I., Nguyen P.D. (2023). Can Craniosynostosis be Diagnosed on Physical Examination? A Retrospective Review. J. Craniofacial Surg..

[81] De Martino L., Mirabelli P., Quaglietta L., Ferrara U.P., Picariello S., De Gennaro D.V., Aiello M., Smaldone G., Aliberti F., Spennato P. (2024). Biobank for craniosynostosis and faciocraniosynostosis, rare pediatric congenital craniofacial disorders: A study protocol. Child’s Nerv. Syst..

[82] Cinalli G., Russo C., Vitulli F., Parlato R.S., Spennato P., Imperato A., Quarantelli M., Covelli E., Aliberti F. (2022). Changes in venous drainage after posterior cranial vault distraction and foramen magnum decompression in syndromic craniosynostosis. J. Neurosurg. Pediatr..

[83] Doerga P.N., Lequin M.H., Dremmen M.H.G., den Ottelander B.K., Mauff K.A.L., Wagner M.W., Hernandez-Tamames J.A., Versnel S.L., Joosten K.F.M., van Veelen M.-L.C. (2020). Cerebral blood flow in children with syndromic craniosynostosis: Cohort arterial spin labeling studies. J. Neurosurg. Pediatr..

[84] Florkow M.C., Willemsen K., Mascarenhas V.V., Oei E.H.G., van Stralen M., Seevinck P.R. (2022). Magnetic Resonance Imaging Versus Computed Tomography for Three-Dimensional Bone Imaging of Musculoskeletal Pathologies: A Review. J. Magn. Reson. Imaging.

[85] Low X.Z., Lim M.C., Nga V., Sundar G., Tan A.P. (2021). Clinical application of “black bone” imaging in paediatric craniofacial disorders. Br. J. Radiol..

[86] Tan A.P. (2019). MRI Protocol for Craniosynostosis: Replacing Ionizing Radiation–Based CT. Am. J. Roentgenol..

[87] Eshraghi Boroojeni P., Chen Y., Commean P.K., Eldeniz C., Skolnick G.B., Merrill C., Patel K.B., An H. (2022). Deep-learning synthesized pseudo-CT for MR high-resolution pediatric cranial bone imaging (MR-HiPCB). Magn. Reson. Med..

[88] Goh C.Y.K., Mohamed Ali P.S., Lee K.H.C., Sim F.Y., Chong L.R. (2025). 3D MRI with CT-like bone contrast (3D-BONE): A pictorial review of clinical applications. Acta Radiol..

[89] Leonhardt Y., Kronthaler S., Feuerriegel G., Karampinos D.C., Schwaiger B.J., Pfeiffer D., Makowski M.R., Koerte I.K., Liebig T., Woertler K. (2022). CT-like MR-derived Images for the Assessment of Craniosynostosis and other Pathologies of the Pediatric Skull. Clin. Neuroradiol..

[90] Eley K.A., Delso G. (2020). Automated 3D MRI rendering of the craniofacial skeleton: Using ZTE to drive the segmentation of black bone and FIESTA-C images. Neuroradiology.

[91] Lethaus B., Gruichev D., Gräfe D., Bartella A.K., Hahnel S., Yovev T., Pausch N.C., Krause M. (2020). “Black bone”: The new backbone in CAD/CAM-assisted craniosynostosis surgery?. Acta Neurochir..

[92] Ducis K., Florman J.E., Rughani A.I. (2016). Appraisal of the Quality of Neurosurgery Clinical Practice Guidelines. World Neurosurg..

[93] Martens J., de Jong G., Rovers M., Westert G., Bartels R. (2018). Importance and Presence of High-Quality Evidence for Clinical Decisions in Neurosurgery: International Survey of Neurosurgeons. Interact. J. Med. Res..

[94] Kocabalkanli C., Fenton R., Gaetani S., Aalamifar F., Linguraru M.G., Seifabadi R. (2024). Accuracy of Cranial Shape Measurements Using Smartphones: A Prospective Study. Cleft Palate Craniofacial J..

[95] Colletti G., Di Bartolomeo M., Negrello S., Geronemus R.G., Cohen B., Chiarini L., Anesi A., Femino R., Mariotti I., Levitin G.M. (2023). Multiple General Anesthesia in Children: A Systematic Review of Its Effect on Neurodevelopment. J. Pers. Med..

[96] Grabowski J., Goldin A., Arthur L.G., Beres A.L., Guner Y.S., Hu Y.Y., Kawaguchi A.L., Kelley-Quon L.I., McAteer J.P., Miniati D. (2021). The effects of early anesthesia on neurodevelopment: A systematic review. J. Pediatr. Surg..

[97] Xiao A., Feng Y., Yu S., Xu C., Chen J., Wang T., Xiao W. (2022). General anesthesia in children and long-term neurodevelopmental deficits: A systematic review. Front. Mol. Neurosci..

[98] Moltoni G., Lucignani G., Sgrò S., Guarnera A., Rossi Espagnet M.C., Dellepiane F., Carducci C., Liberi S., Iacoella E., Evangelisti G. (2024). MRI scan with the “feed and wrap” technique and with an optimized anesthesia protocol: A retrospective analysis of a single-center experience. Front. Pediatr..

[99] Liu J., Elkhill C., LeBeau S., French B., Lepore N., Linguraru M.G., Porras A.R. (2022). Data-driven Normative Reference of Pediatric Cranial Bone Development. Plast. Reconstr. Surg. Glob. Open.

[100] Robertson E., Boulanger P., Kwan P., Louie G., Aalto D. (2024). Improving Cranial Vault Remodeling for Unilateral Coronal Craniosynostosis—Introducing Automated Surgical Planning. Craniomaxillofacial Trauma. Reconstr..

[101] Sharma D., Holden A.M., Nezamivand-Chegini S. (2025). Augmented Reality Integration in Surgery for Craniosynostoses: Advancing Precision in the Management of Craniofacial Deformities. J. Clin. Med..

[102] Soldozy S., Yağmurlu K., Akyeampong D.K., Burke R., Morgenstern P.F., Keating R.F., Black J.S., Jane J.A., Syed H.R. (2021). Three-dimensional printing and craniosynostosis surgery. Child’s Nerv. Syst..

[103] Yoshida M.M., Freitas A.L.P.d., Carvalho Júnior J.d.C., Souza J.S.d., Baptista V.S., Ferreira L.M. (2025). Holographic model of craniosynostosis for HoloLens. Acta Cirúrgica Bras..

